# Intermediate Disturbances Enhance Microbial Enzyme Activities in Soil Ecosystems

**DOI:** 10.3390/microorganisms12071401

**Published:** 2024-07-11

**Authors:** Hojeong Kang, Sunghyun Kim, Keunyea Song, Min-Jung Kwon, Jaehyun Lee

**Affiliations:** 1 School of Civil and Environmental Engineering, Yonsei University, Seoul 03722, Republic of Korea; jaehyunlee@kist.re.kr; 2 Smithsonian Environmental Research Center, Edgewater, MD 21037, USA; skim@si.edu; 3 Department of Ecology, State of Washington, Lacey, WA 98504, USA; keunyea@gmail.com; 4 Institute of Soil Science, Universität Hamburg, 20146 Hamburg, Germany; minjung.kwon86@gmail.com

**Keywords:** Intermediate Disturbance Hypothesis, extracellular enzyme activities, bactericide

## Abstract

The Intermediate Disturbance Hypothesis (IDH) posits that maximal plant biodiversity is attained in environments characterized by moderate ecological disturbances. Although the applicability of the IDH to microbial diversity has been explored in a limited number of studies, there is a notable absence of experimental reports on whether soil microbial ‘activity’ demonstrates a similar response to the frequency or intensity of environmental disturbances. In this investigation, we conducted five distinct experiments employing soils or wetland sediments exposed to varying intensities or frequencies of disturbances, with a specific emphasis on disturbances associated with human activity, such as chemical contamination, hydrologic changes, and forest thinning. Specifically, we examined the effects of bactericide and heavy metal contamination, long-term drainage, tidal flow, and thinning management on microbial enzyme activities in soils. Our findings revealed that microbial enzyme activities were highest at intermediate disturbance levels. Despite the diversity in experiment conditions, each trial consistently demonstrated analogous patterns, suggesting the robustness of the IDH in elucidating microbial activities alongside diversity in soils. These outcomes bear significant implications for ecological restoration and management, as intermediate disturbance may expedite organic matter decomposition and nutrient cycles, crucial for sustaining ecosystem services in soils.

## 1. Introduction

The Intermediate Disturbance Hypothesis (IDH) posits that maximum species diversity is attained when ecological disturbances occur at an intermediate frequency or intensity, avoiding extremes of rarity or frequency. Although its efficacy has faced scrutiny from some researchers [1], the IDH has undergone thorough empirical and theoretical scrutiny by others [2,3,4,5]. Empirical studies have extensively examined how disturbances, such as forest fires, deforestation, diseases, grazing, and flooding, impact plant diversity [6]. Conventional IDH asserts that optimal plant diversity is achieved at intermediate disturbance levels. Low frequency or the absence of disturbance fosters resource monopolization by a few dominant species, yielding low diversity, while high disturbance levels favor colonizing-resistant species only, again leading to reduced diversity. Hence, intermediate disturbance levels are theorized to maximize species diversity. Although initially proposed to elucidate plant diversity, the IDH has found application in diverse ecosystems, including invertebrate diversity in tropical streams [7] and biodiversity along salinity gradients [8].

Microorganisms in soils are pivotal in organic matter decomposition and mineralization at local and global scales. While extensive research has elucidated their role in global nutrient cycles and environmental sustainability [9], microbial ecology has diverged somewhat from plant or animal ecology in theoretical development. This divergence is attributed to the inherent challenges in observing microorganisms in their natural environments and the reductionist approach commonly employed by microbiologists [10]. In this context, the IDH presents an intriguing theoretical framework for better understanding the connection between environmental factors, microbial diversity, and microbial activity.

Recent research has sought to integrate the IDH into microbial ecology to interpret variations in microbial diversity in response to environmental disturbances. Studies exploring diverse disturbances, including mercury contamination [11], viral infections in marine bacteria [12], spring ecosystems [13], and animal trampling [14], have confirmed that microbial community structure aligns with the IDH, exhibiting maximal diversity under mid-range disturbances [15].

However, previous investigations have predominantly focused on microbial diversity, overlooking the significance of microbial activities in ecological processes [16]. Given the pivotal role of microbial activities in ecosystem processes such as biogeochemical cycles, nutrient cycles, and virulence of medically important microbes, understanding their response to disturbances is crucial [15]. Additionally, microbial diversity does not always correlate directly with activity or process rates due to potential redundancy among microbial taxa [16]. While various approaches have been proposed to assess microbial activities, soil enzymes have emerged as a popular method to assess microbial activity in soils due to their central role in decomposition processes [17] and operational advantage. They mainly originate from microorganisms and function either freely or in association with soil organic matter or minerals, contributing to the mineralization of organic matter. Soil enzymes have been widely assessed across various ecosystems as they indicate decomposition rates of organic matter, rate-limiting steps in nutrient cycles, and overall ecosystem health, and serve as an index of restoration. Consequently, they are key indicators in environmental microbial ecology. In particular, linking IDH and enzyme activity could be valuable in agricultural, food, and medical microbiology applications, as disturbances in these areas could either activate or deactivate target microorganisms in a given condition. 

In this context, the purpose of this study is to elucidate the effects of disturbance in-tensity and frequency on microbial enzyme activities using samples from forest soils, marsh sediments, tropical peatlands, and coastal mud flats. The hypothesis is that microbial activities will peak under mid-range disturbance, extending the Intermediate Disturbance Hypothesis to microbial activities. The study focuses on common human activities, such as chemical contamination, hydrologic alteration, and forest thinning, as the sources of disturbance.

## 2. Materials and Methods

The IDH was tested with five different types of soil and wetland sediments, which were exposed to different environmental disturbances: chemical contaminations (i.e., bacteriocide additions and cadmium additions), hydrologic changes (i.e., long-term drainage and tidal wave), and forest thinning (i.e., control on the number of trees per unit area) (Table 1).

### 2.1. Chemical Contaminants

Two experiments were conducted to examine the effects of chemical contaminations (i.e., bacteriocide or cadmium additions). Dehydrogenase and β-glucosidase activities in forest soils which were exposed to different frequencies of bacteriocide additions were compared. We collected soil samples from Autumn Hill Reservation (40°22′ N, 74°38′ E), Princeton, NJ, USA. The main vegetation consists of *Quercus bicolar* and *Acer Saccharinum*, the organic matter content of soil is 9.8%, and soil texture is sandy loam. Soils were collected to 10 cm depth from the surface and sieved with a 2 mm sieve to remove root debris and large particles prior to being placed in vials. In each vial, 1 g of soil was placed and 0.15 mL of water or benzalkonium chloride solution was added daily for 10 days. Each group was treated with water only (control), twice with benzalkonium chloride (low frequency), four times with benzalkonium chloride (mid frequency) or ten times with benzalkonium chloride (high frequency). Three replicate samples were prepared for each treatment. Dehydrogenase activity was measured on day 10 using INT (Iodonitrozotetrazolium) as a model substrate, while β-glucosidase was measured using MUF-β-glucopyranoside [18]. For the dehydrogenase assay, 5 g of soil was incubated with 10 mL of INT solution for 1 h, followed by termination of the reaction by centrifugation. β-glucosidase activity was measured using 1 g of soil amended with 400 µmoles of MUF-β-glucopyranoside. The fluorescence of the product was measured at excitation and emission wavelengths of 360 nm and 460 nm, respectively.

To assess effects of cadmium (Cd) addition, marsh sediment was exposed to different concentrations of Cd. Two hundred grams of fresh marsh sediment collected from a Siwha marsh (37°15′ N, 126°51′ E) in Korea was placed in a jar, and water was added to the surface. The microcosm was amended with cadmium of 0, 1, 10, and 10^2^ mg kg^−1^ and incubated for 10 days at 15 °C. Two grams of sub-samples were collected at day 1 and day 10, and dehydrogenase and β-glucosidase activities were determined using INT and MUF-β-glucopyranoside as model substrates, respectively.

### 2.2. Hydrological Changes

Effects of water level fluctuations were assessed with tropical peats and coastal mud flat. Peat cores were collected from three locations at different water regimes in central Kalimantan (1°88′ N, 113°53′ E), Indonesia. The sites were (1) a ditched site with at least a six-year drainage history, (2) an edge site at the margin of a pristine area with an intermediate water regime, and (3) an undisturbed site located near the center of a pristine peatland with no history of water drainage. The partial data from the sites have already been published [18]. The soils are peat with organic matter content of 92.7%, and detailed information about the site has previously been reported [19]. The activities of three enzymes, β-glucosidase, N-acetylglucosaminidse, and β-xylosidase, were measured using methylumbelliferyl compounds as a model substrate. To reveal effects of tidal flows, we collected intact mud-flat sediment cores and sea water samples at Kanghwa area (37°26′ N, 126°27′ E) in South Korea. The soil texture was silty clay loam and organic matter content was 20%. Collected sediment cores with three replicates were then exposed to different frequencies of tidal flows: high frequency (every 12 h), mid frequency (every 24 h), and low frequency (every 48 h) over a five-week period. Filtered sea water (salinity of 30 ppt) was introduced to each treatment using a peristaltic pump. β-glucosidase, N-acetylglucosaminidse, and phosphatase activities were measured at the end of the incubation using MUF compounds as model substrates.

### 2.3. Thinning Management

We collected soil samples from forest floors with different intensities of thinning. The study site was Gwang-neung forestry research station of Korea Forestry Agency (37°74′ N, 127°15′ E), with pine trees (*Pinus koraiensis*) the dominant species. Samples were collected from four different treatments, which were control (no thinning), 700 trees ha^−1^, 600 trees ha^−1^, and 500 trees ha^−1^, which is in the order of disturbance intensity. Surface soil samples (0–10 cm) were collected in summer and β-glucosidase, cellobiohydrolase, N-acetylglucosaminidase, and β-xylosidase activities were determined by an aforementioned method.

### 2.4. Statistical Analysis

Significant differences among diverse disturbance intensities were determined by one-way ANOVA followed by Tukey’s test. Significant differences were reported at *p* < 0.05.

## 3. Results

**Figure 1:**
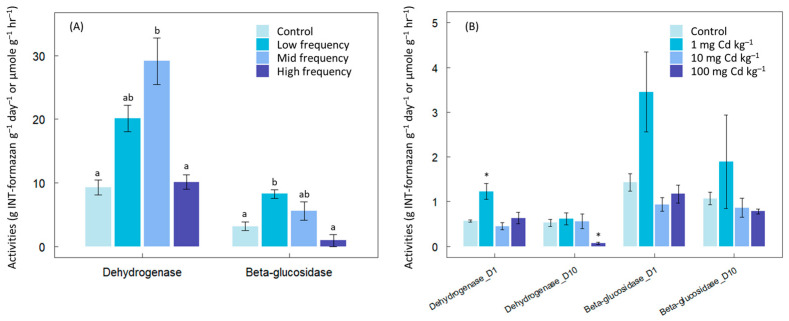
Effects of toxic chemicals on dehydrogenase and β-glucosidase activity: (**A**) Effects of addition frequency of benzalkonium chloride activity in temperate forest soils. Data labeled with different letters are significantly different at *p* < 0.05 (**B**) Effects of additions of different amount of cadmium in a marsh sediment. D1 and D10 denote incubation period of 1 day and 10 days, respectively. Data labelled with * are significantly different at *p* < 0.05 at each enzyme and incubation period.

The addition of toxic chemicals resulted in increased enzyme activities across both chemical types and soil variations. In the initial experiment, dehydrogenase and β-glucosidase activities rose with increasing benzalkonium chloride addition up to the mid-frequency level (Figure 1A). However, enzyme activity returned to control levels at the highest frequency of addition. Notably, the mid-frequency benzalkonium chloride addition exhibited enzyme activities over three times higher than those observed in the control or high-frequency addition conditions. Cadmium additions showed a similar trend, whereby low disturbance levels increased enzyme activities. Specifically, dehydrogenase activity significantly increased with 1 mg kg^−1^ addition, albeit disappearing by day 10 (Figure 1B). In this system, the intermediate levels might exist somewhere between 1 and 10 mg Cd kg^−1^, which was not clearly identified in this experiment. Remarkably, additions exceeding 100 mg kg^−1^ dramatically decreased dehydrogenase activities by day 10, indicating the toxic impact of Cd on microbial metabolic activity in sediment.

**Figure 2:**
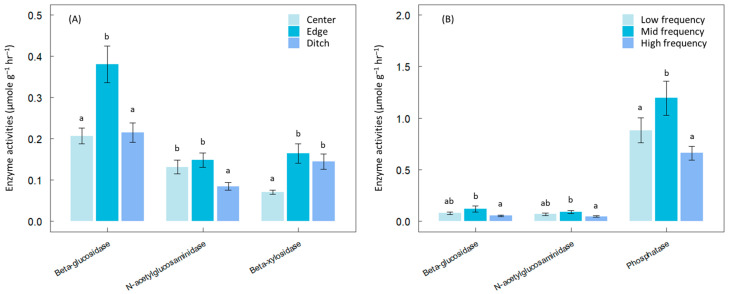
Effects of water level fluctuations on enzyme activities in (**A**) tropical peatland (long-term effects) and (**B**) mud-flat sediment (short-term effects). Data labeled with different letters are significantly different at *p* < 0.05.

While the initial and subsequent experiment underscored the significance of chemical contamination as a short-term disturbance, the third experiment highlighted the enduring legacy of hydrological disturbance, revealing a similar pattern. Enzyme activities peaked in edge areas compared to pristine or six-year drained sites (Figure 2A). Similarly, alterations in tidal flow frequency mirrored IDH patterns, with enzyme activities highest under mid-range tidal flow across β-glucosidase, N-acetylglucosaminidase, and phosphatase (Figure 2B).

**Figure 3:**
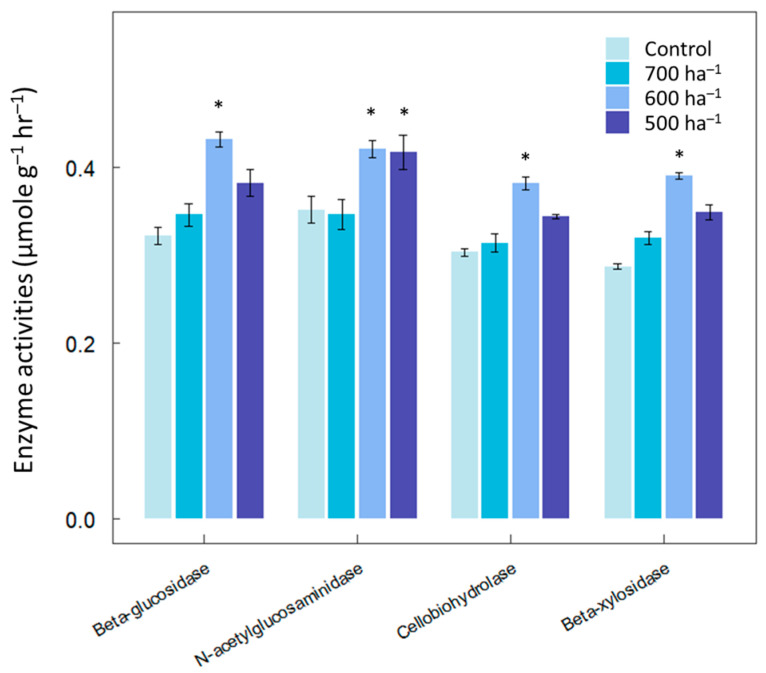
Effects of thinning on enzyme activities in *Pinus koraiensis* forests. Data labelled with asterisks are significantly different from others at *p* < 0.05.

The final dataset aimed to evaluate the impact of thinning on soil enzyme activities. All four enzymes exhibited significantly higher activities at a mid-range intensity of 600 trees ha^−1^ compared to the control or 500 trees ha^−1^ treatment (Figure 3). Both 600 and 700 trees ha^−1^ treatments demonstrated higher N-acetylglucosaminidase activities compared to the other two treatments.

**Figure 4:**
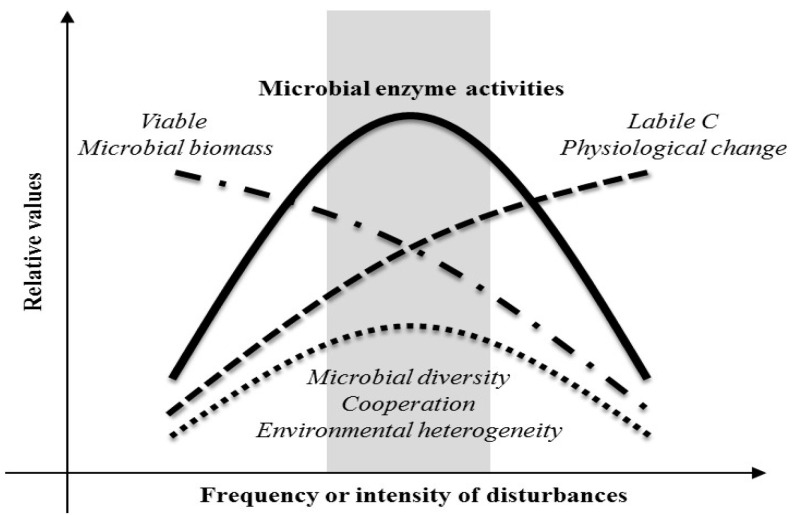
A conceptual model for mechanism of the IDH in relation to microbial activities in soil systems based on the findings of this study.

The higher activities observed at mid-intensity disturbance in our study may be attributed to several mechanisms (Figure 4). High disturbance levels may inhibit microbial activities but induce faster turnover of microbial cells, providing labile carbon sources for remaining viable cells and resulting in overall higher activity. Intermediate disturbance may also promote higher microbial diversity, increasing support for specific groups of microorganisms with higher activities. Additionally, cooperation among different taxa of microorganisms may peak at intermediate disturbance, leading to higher enzyme activities. Certain stresses induced by intermediate disturbance may modify physiological responses of existing microorganisms, resulting in enzyme induction or increased metabolic rates. Moreover, certain disturbances at mid-range intensity may maximize nutrient availability, oxygen penetration, light intensity, or understory vegetation, facilitating higher enzyme activities.

## 4. Discussion

This study represents a pioneering effort to establish a link between the IDH and microbial enzyme activities in soil ecosystems, building upon similar approaches applied in aquatic ecosystems [20]. Soil microbial activities play a fundamental role in numerous ecological functions, including nutrient cycling and organic matter decomposition, through the release of extracellular enzymes. This process is critical for sustaining life on Earth, as organic matter decomposition provides essential nutrients for plants and energy for microbes, and aids in pollutant removal from the environment.

Previous research has shown that benzalkonium chloride addition leads to progressively stronger inhibition of dehydrogenase [21]. However, these studies typically employed higher chemical dosages and pure cultures of microorganisms, limiting observations of intermediate disturbance. Contrary to these findings, our study observed higher dehydrogenase and β-glucosidase activity resulting from intermediate frequencies of benzalkonium addition. This increase may be explained by several mechanisms. Benzalkonium chloride could act as a selective pressure, fostering the development of a resistant or benzalkonium-chloride-degrading microbial community [22]. Additionally, the destruction of a portion of microbes may provide labile carbon for remaining microorganisms, stimulating active metabolism [23]. Soil bacteria may also enhance their metabolic rates to cope with physiological stress induced by the disinfectant. Furthermore, cooperation among different microbial groups may peak at intermediate disturbance, leading to maximal biomass at mid-frequency disturbance, as suggested by Brockhurst et al. [24]. Similar mechanisms may account for the elevated activities of dehydrogenase and β-glucosidase under 1 mg Cd Kg^−1^ addition compared to other concentrations, as was noted in a Cu application experiment [25].

Peatlands typically develop under water-logged and ombrotrophic conditions where water is supplied solely by precipitation, resulting in low decomposition rates due to oxygen deprivation and low pH [26]. Water level fluctuations in peatlands can activate enzyme activities and accelerate decomposition by allowing oxygen penetration. Conversely, severe or prolonged drainage may limit microbial activities by restricting water supply. Coastal wetlands may experience inhibited enzyme activities due to lack of oxygen penetration and high salinity during frequent flooding, while extended exposure to air can lead to soil drying and immense stress for microorganisms. Our findings support the notion that intermediate intensity or frequency of hydrological disturbances activates enzyme activities in soils compared to absence or too-frequent flooding [27].

Thinning, a common forest management technique, involves selective removal of suppressed trees to enhance timber production [28]. Previous studies have suggested that intermediate thinning sites exhibit higher microbial biomass and enzyme activity than non-thinned control or heavily thinned sites, similar to our finding. For example, Wu et al. [29] found higher enzyme activities in light- or mid-intensity thinning sites compared to control or high-intensity thinning sites in *Larix* forests in China. Similarly, Zhou et al. [30] conducted a meta-analysis across global forests, revealing higher carbon-related oxidase and hydrolase activities in moderately thinned forests. Thinning can increase soil temperature and moisture content by reducing canopy cover, thereby enhancing enzyme activities. Additionally, mid-range thinning may promote understory vegetation growth, compensating for reduced carbon supply from leaf litter and fine roots.

In addition to extending the IDH concept to microbial activities in soil ecosystems, our findings have implications for other microbial systems, such as medical and food microbiology. Incomplete sterilization may induce stronger virulence or activity of harmful microorganisms under medium-range disturbance conditions. This concept also applies to ecosystem restoration and conservation [31], where appropriate disturbance levels can enhance soil microbial process rates. Proper disturbance intensity can accelerate ecosystem restoration or maintenance of critical functions, particularly relevant for wetland restoration, where specific water level fluctuations are necessary to maintain biogeochemical processes and ecosystem functions.

## 5. Conclusions

Our study demonstrates that the IDH, originally developed for plant ecology, can be applied to microbial ecology, particularly concerning microbial activities in soil ecosystems. This finding underscores the potential for ecological theory-driven research to provide valuable insights into the complex interactions between microorganisms and their environment. Future studies employing high-resolution community analysis and chemical analysis may elucidate the underlying mechanisms driving microbial activity patterns under different disturbance regimes. Overall, this study contributes to our understanding of microbial ecology and emphasizes the importance of considering microbial communities in ecosystem management and conservation efforts.

## Figures and Tables

**Table 1:** Types of disturbances, target enzymes, and sources of the samples.

Disturbance		Target Enzymes	Sample Sources
Chemical contaminations	Bacteriocide	Dehydrogenase β-glucosidase	Forest soil
	Cd addition	Dehydrogenase, β-glucosidase	Marsh sediment
Hydrological stress	Drought	β-glucosidase N-acetylglucosaminidase β-xylosidase	Tropical peatland
	Tidal flow	β-glucosidase N-acetylglucosaminidase β-xylosidase	Mud-flat sediment
Management	Thinning	β-glucosidase Cellobiohydrolase N-acetylglucosaminidase β-xylosidase	Forest soils

## Data Availability

The original contributions presented in the study are included in the article, further inquiries can be directed to the corresponding author.
